# The Circadian Nobiletin-ROR Axis Suppresses Adipogenic Differentiation and IκBα/NF-κB Signaling in Adipocytes

**DOI:** 10.3390/nu15183919

**Published:** 2023-09-09

**Authors:** Eunju Kim, Kazuaki Mawatari, Seung-Hee Yoo, Zheng Chen

**Affiliations:** 1 Department of Biochemistry and Molecular Biology, McGovern Medical School, The University of Texas Health Science Center at Houston (UTHealth Houston), Houston, TX 77030, USA; seung-hee.yoo@uth.tmc.edu; 2 Department of Preventive Environment and Nutrition, Institute of Biomedical Sciences, Tokushima University Graduate School, Kuramoto-cho 3-18-15, Tokushima 770-8503, Japan; mawatari@tokushima-u.ac.jp

**Keywords:** Nobiletin, retinoic acid receptor-related orphan receptors (RORs), circadian clock, adipocytes, lipid, IκBα/NF-κB, 3T3-L1, obesity

## Abstract

Obesity is a known risk factor for metabolic diseases and is often associated with chronic inflammation in adipose tissue. We previously identified the polyethoxylated flavonoid Nobiletin (NOB) as a circadian clock modulator that directly binds to and activates the ROR receptors in the core oscillator, markedly improving metabolic fitness in obese mice. Here, we show that NOB enhanced the oscillation of core clock genes in differentiated 3T3-L1 adipocytes, including ROR target genes such as *Bmal1*, *Cry1*, *Dec1*, and *Dec2*. NOB inhibited lipid accumulation in 3T3-L1 and SVF cells, concomitant with the dysregulated circadian expression of adipogenic differentiation-related genes including *Cebpb*, *Pparg*, *Lpl*, *Scd1*, and *Fas.* Importantly, RORα/RORγ double knockdown in 3T3-L1 cells (Ror DKD) significantly attenuated the effects of NOB on circadian gene expression and lipid accumulation. Furthermore, whereas NOB upregulated the expression of IκBα, a target of RORs, to inhibit NF-κB activation and proinflammatory cytokine expression, Ror DKD cells exhibited a heightened activation of the NF-κB pathway, further indicating a requisite role of RORs for NOB efficacy in adipocytes. Together, these results highlight a significant regulatory function of the NOB–ROR axis in the circadian expression of clock and clock-controlled genes in adipocytes, thereby governing adipogenic differentiation, lipogenesis, and inflammation.

## 1. Introduction

Obesity constitutes a significant health challenge worldwide, strongly associated with the prevalence of various debilitating diseases including type 2 diabetes, cardiovascular diseases, hypertension, and cancer [1]. Adipocytes, the cells specialized in storing fat in the adipose tissue, are known to expand in obesity [2]. Various studies have indicated that hindering adipogenesis and limiting fat storage could offer therapeutic efficacies to combat obesity-related issues [2,3].

The immortal NIH 3T3-L1 cell line is a widely used in vitro model for studying adipogenesis and lipogenesis. Under controlled laboratory conditions, these cells can differentiate from preadipocytes to mature adipocytes. During the differentiation of 3T3-L1 cells, the expression and activity of several transcription factors, such as CCAAT/enhancer binding proteins (C/EBPs) and peroxisome proliferator-activated receptors (PPARs), are activated to control adipocyte differentiation and maintain adipocyte physiology [4,5]. These factors also regulate the target gene expression associated with lipid accumulation, including lipoprotein lipase (*Lpl*), stearoyl-CoA desaturase-1 (*Scd1*), and fatty acid synthase (*Fas*) [6], enabling the storage of excess energy as triglycerides in adipocytes and contributing to fat accumulation in the adipose tissue [7,8].

Obesity is linked to the persistent activation of inflammatory pathways in both adipocytes and macrophages present in or entering the adipose tissue. In addition to being a storage site for fat, the adipose tissue also functions as an endocrine organ, releasing various adipokines, including inflammatory cytokines, into the bloodstream [9,10]. Inflammatory pathways such as nuclear factor-κB (NF-κB) signaling are upregulated in obese adipose tissue, leading to an increased expression of downstream cytokines such as tumor necrosis factor-a (TNFα), interleukin-6 (IL6), interleukin-1beta (IL1β), among others [10,11,12]. These cytokines contribute to local inflammation and trigger the secretion of additional pro-inflammatory substances, establishing a deleterious cycle of the inflammatory response [13].

Citrus flavonoids display varying degrees of methoxylation and glycosylation, and offer a wide range of beneficial effects in health and diseases. Polymethoxylated flavonoids (PMFs), such as Nobiletin (NOB) and its close analog Tangeretin, exhibit improved pharmacokinetic properties compared to their less methoxylated counterparts [14,15,16]. Notably, NOB elicits numerous protective effects, including anti-obesity, anti-cancer, and anti-inflammatory properties [17,18,19,20,21,22]. In 3T3-L1 cells, initial evidence indicates that NOB suppresses the differentiation of preadipocytes into mature adipocytes [23,24,25], prevents an excessive accumulation of adipocytes via induction of browning [24], and exerts anti-inflammatory effects by reducing the production of proinflammatory cytokines and markers [26]. However, the mechanistic pathways and direct protein targets underlying NOB effects in adipocytes are poorly understood.

The growing literature points to the circadian clock as a key cellular target for NOB. Specifically, NOB has been found to enhance the amplitude of circadian rhythms, the daily rhythmic processes that occur in the body [27]. In situations where the circadian amplitude is diminished, such as in metabolic disorders, aging, and Alzheimer’s disease (AD), mice treated with NOB showed significant improvements [28,29,30,31]. In addition, NOB exhibited an anti-inflammatory effect in AD and cancer mouse models by modulating NF-κB nuclear translocation and reducing pro-inflammatory cytokines [22,30]. These finding suggest that NOB may play a crucial role as a clock modifier in metabolism and physiology, promoting overall fitness throughout lifetime. Importantly, competitive filter-binding analysis, a gold standard assay for compound–protein interactions, revealed that NOB directly interacts with and activates retinoic acid receptor-related orphan receptors (RORs, specifically the α and γ subtypes), nuclear receptors in the core oscillator serving to govern the robust circadian expression of core clock genes, as well as a myriad other target genes [22,27,28,32,33]. This provides a mechanistic explanation for NOB’s ability to enhance circadian rhythms and promote clock-associated physiological functions. Given the critical role of the clock to govern energy homeostasis including in the adipose tissues [34,35], we examined whether RORs and the circadian oscillator are involved in mediating the anti-obesity effects of NOB. Our mechanistic and functional analyses uncovered a powerful anti-obesity mechanism involving the NOB–ROR axis that regulates circadian gene expression, particularly the IκBα/NF-κB pathway. These findings highlight a promising and actionable approach to combat obesity.

## 2. Materials and Methods

### 2.1. Cell Culture

The 3T3-L1 preadipocyte and stromal vascular fraction (SVF) cell lines were obtained from Dr. Hyun Ho Choi and Dr. Kai Sun at UTHealth Houston, respectively. Dulbecco’s Modified Eagle’s Medium (DMEM, GenDEPOT, Baker, TX, USA) supplemented with 10% fetal bovine serum (FBS, GenDEPOT, TX, USA) and 100 mg/mL of penicillin/streptomycin (GenDEPOT) was used to culture 3T3-L1 and SVF cells at 37 °C in a 5% CO_2_ incubator until 90% confluence. Confluent cells were maintained in differentiation induction medium consisting of 10 mg/mL of insulin (Sigma, St. Louis, MO, USA), 0.25 mM dexamethasone (Sigma), and 0.5 mM 3-isobutyl-1-methylxanthine (IBMX, Sigma) in 10% FBS-contained DMEM for two days, followed by maturation medium containing 10% FBS and 10 mg/mL of insulin for six days. Nobiletin (NOB) (Selleckchem, Houston, TX, USA) was first added at 10 or 20 μM concentrations to differentiation induction media and maintained in subsequent media. 

To generate Rora/Rorc double knockdown cell lines (Ror DKD), we carried out CRISPR as previously described [36]. The gRNAs, sense and antisense, were designed using the https://crispr.dbcls.jp/ program (accessed on 12 January 2020) and cloned into the BsmB1 site of the GeCKO vector [37] for 3T3-L1 transfection followed by puromycin selection. Clones with Ror DKD were selected based on expression analyses.

### 2.2. Bioluminescence Measurement Using 3T3-L1 Cells

To monitor circadian rhythms in 3T3-L1, we transfected and generated clones with stable expression of the *Bmal1:Luciferase* reporters [38]. Cells were cultured on 35 mm plates and were synchronized with 200 nM dexamethasone (Dex; Sigma) for 1 h. After DMSO or NOB (10 μM) containing recording media [39] were added, the dishes were sealed with vacuum silicon grease and bioluminescence was measured in Kronos Dio AB-2550 (ATTO, Tokyo, Japan) for continuous bioluminescence monitoring. The data were detrended using a first-order polynomial, and then best-fit to a sine wave estimated by a Levenberg–Marquardt algorithm for measurement of circadian parameters in the CircaCompare software (Version 1.0.0; R package) [40].

### 2.3. Hematoxylin and Eosin (H&E) Staining

Control and NOB-treated cells were subjected to differentiation for 8 days for parental 3T3-L1 cells (denoted hereafter as 3T3-L1) and 6 days for Ror DKD cells. Subsequently, the cells were rinsed using phosphate-buffered saline (PBS, GenDEPOT), fixed with 4% Paraformaldehyde at room temperature for 15 min, and washed three times more with deionized water. Next, 3% triton X-100 was added for 20 min to allow the stain to enter the nucleus. After washing with 3% triton X-100, filtered Gill3 Hematoxylin (Thermo Scientific, Waltham, MA, USA) was added for 2–3 min. The cells were washed three times with ddH_2_O and then subjected to staining with Eosin Y (Thermo Scientific) for a few seconds. After thorough washing with an adequate amount of ddH2O, the cells were mounted using a mounting solution (Cytoseal, Thermo Scientific).

### 2.4. Oil Red O Staining

Vehicle- and NOB-treated cells were differentiated for 8 days for 3T3-L1 cells and 6 days for Ror DKD cells, followed by washing with phosphate-buffered saline (PBS, GenDEPOT), fixation with 10% formalin for 1 h at room temperature, and washing three times more with deionized water. Cells were treated with a mixture containing 0.6% Oil Red O dye in isopropanol, combined with water in a 6:4 ratio. This mixture was applied to the cells for 20 min. Afterward, the stained cells were thoroughly rinsed three times using deionized water and then left to air-dry completely. Images were captured under a microscope (BX60 Olympus, Tokyo, Japan). Oil Red O stained areas were calculated by using ImageJ software (version 1.53e) to determine the percent of positive lipid areas. Briefly, the images were transformed into 8-bit grayscale images and a threshold was defined by the range of the RGB color.

### 2.5. Real-Time PCR Analysis

RT-qPCR analysis was conducted as previously described with minor modifications [22]. For real-time qPCR analysis, cells were synchronized with 200 nM dexamethasone (Sigma) at 6–8 days after differentiation (8 days; 3T3-L1, 6 days; Ror DKD). Cells were harvested every 4 h for 24 h (6 time points). Total RNA was isolated from differentiated 3T3-L1 cells using PureXtract RNAsol reagent (GenDEPOT). cDNA was synthesized with a cDNA synthesis kit (GenDEPOT). mRNA gene expressions were measured by using QuantStudio 7 Flex system (Applied Biosystems, Waltham, MA, USA). *Gapdh* was used as the loading control. Primer sequences are listed in Table 1.

### 2.6. Western Blot Analysis

Western blotting was performed as described previously [22]. Briefly, 6–8 days after differentiation (8 days; 3T3-L1, 6 days; Ror DKD), differentiated cells were washed with cold PBS and lysed in 0.5% triton X-100-contained HEPES lysis buffer. Protein extracts were loaded by 10–12% SDS polyacrylamide gel electrophoresis and blotted onto a nitrocellulose membrane. Blocking was performed at room temperature for 1 h in TBS-Tween 20 (TBS-T, GenDEPOT) with 5% blocker (Bio-Rad, CA, USA), followed by incubation with the primary antibodies diluted in TBS-T. After washing with TBS-T, the membrane was incubated with horseradish peroxidase-conjugated secondary antibodies for 1–2 h. The protein bands were visualized using a West-Q Pico ECL solution (GenDEPOT). Primary antibodies against the following proteins were used: RORα (ab256799, Abcam); RORγ (sc-293150, Santa Cruz); IκBα (#9242), p65 (#4764), and phospho-p65 (#3033) (Cell Signaling Technology, MA, USA); and GAPDH (Sigma). To quantify relative protein expression, uncalibrated optical density (OD) levels were measured by ImageJ software. Briefly, the blot images were transformed into 8-bit images and calibrated using the Uncalibrated OD function in ImageJ. Subsequently, a consistent selection area was employed to calculate the average intensity of each band.

### 2.7. TNFα Measurement

During differentiation, media samples were collected on days 6 and 8 after differentiation (day 8 for 3T3-L1, and day 6 for Ror DKD). The collected media samples were subjected to TNFα analysis using an enzyme-linked immunosorbent assay (ELISA) kit from R&D Systems (Minneapolis, MN, USA), following the manufacturer’s recommended protocol.

### 2.8. Statistical Analysis

Each experiment was performed in at least triplicate. Data are presented as mean ± SEM. The statistical significance of the difference was analyzed using one-way ANOVA or two-way ANOVA, followed by Tukey’s test. All statistical analyses were performed using GraphPad Prism 9 (Graphpad Software Inc., San Diego, CA, USA). *p* < 0.05 was considered to be statistically significant.

## 3. Results

### 3.1. NOB Modulates Circadian Rhythms of Core Clock Gene Expression

**Figure 1:**
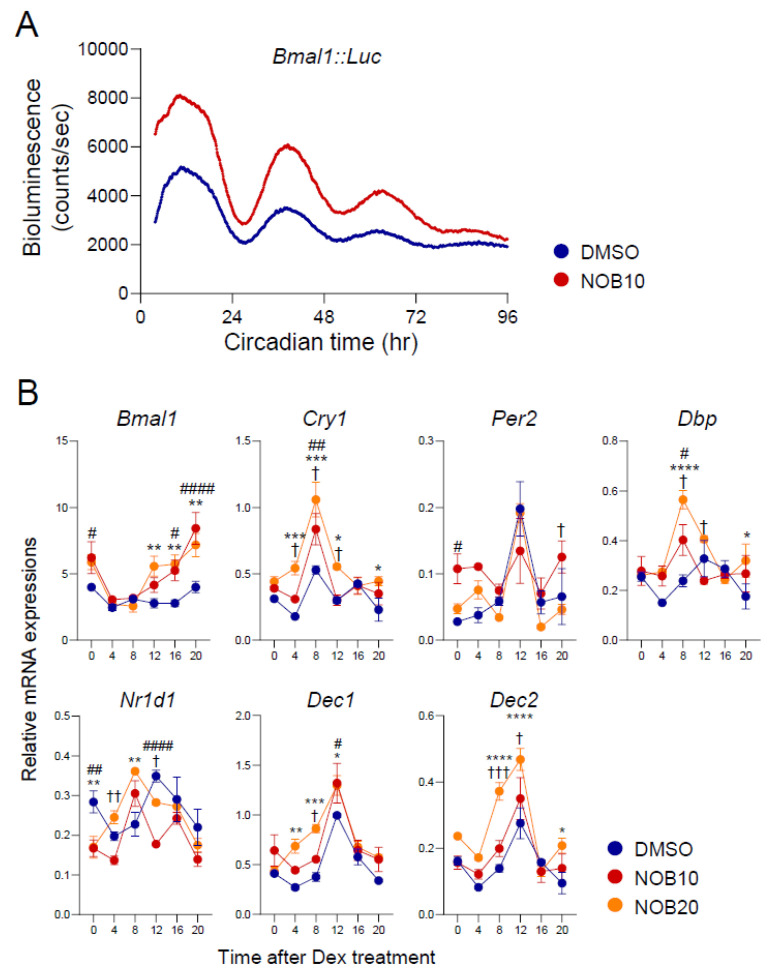
NOB enhances amplitude and alters the phase of circadian gene expression. (**A**) Representative *Bmal1::Luc* bioluminescence recording of *Bmal1::Luc* 3T3-L1 adipocyte cells after Dex-treated synchronization. (**B**) Real-time qPCR analysis of core clock gene expressions in 3T3-L1 cells at 8 days after differentiation. Data are shown as mean ± SEM every 4 h for 24 h (*n* = 3/group/time point). Two-way ANOVA with Tukey’s multiple comparison tests showed significant differences # *p* < 0.05, ## *p* < 0.01, #### *p* < 0.0001, DMSO vs. NOB10; * *p* < 0.05, ** *p* < 0.01, *** *p* < 0.001, **** *p* < 0.0001, DMSO vs. NOB20; † *p* < 0.05, †† *p* < 0.01, ††† *p* < 0.001, NOB10 vs. NOB20.

We first examined how NOB affects adipocyte circadian rhythms using 3T3-L1 cells stably expressing a *Baml1::Luc* reporter [38] (Figure 1A). Specifically, 3T3-L1 reporter adipocyte cells were synchronized with 200 nM Dex followed by NOB treatment at a concentration of 10 µM. Bioluminescence recording showed that NOB-treated cells displayed a robust increase in their circadian amplitude compared to reporter cells treated with DMSO, with a significant difference (*p* = 2.82 × 10^−9^).

We next investigated the effects of NOB on the expression of core clock genes and clock-controlled genes in 3T3-L1 cells. Differentiated 3T3-L1 adipocytes were collected every 4 h after Dex synchronization, followed by DMSO or NOB treatment at either 10 or 20 µM (NOB10 and NOB20). Our prior research established that NOB functions as an agonist to activate RORs in the core clock oscillator, thereby influencing the circadian expression of target genes and impacting metabolic and physiological processes [22,27,28,30,31]. Several clock genes showed significantly different expressions between DMSO- and NOB-treated cells (Figure 1B). For example, expressions of ROR target genes such as *Bmal1* and *Cry1* were markedly enhanced as a result of NOB treatment, in a dose-dependent manner. Likewise, *Dec1* and *Dec2*, which are also ROR target genes encoding transcription factors that inhibit the activity of the circadian transcription factor CLOCK:BMAL1 by direct binding [41], were similarly enhanced by NOB treatment, especially in NOB20. Furthermore, the clock output gene *Dbp* was activated by NOB, consistent with a role of NOB to enhance circadian gene expression in 3T3-L1 adipocytes.

### 3.2. NOB Inhibits Lipid Accumulation in Differentiated 3T3-L1 and SVF Cells

**Figure 2:**
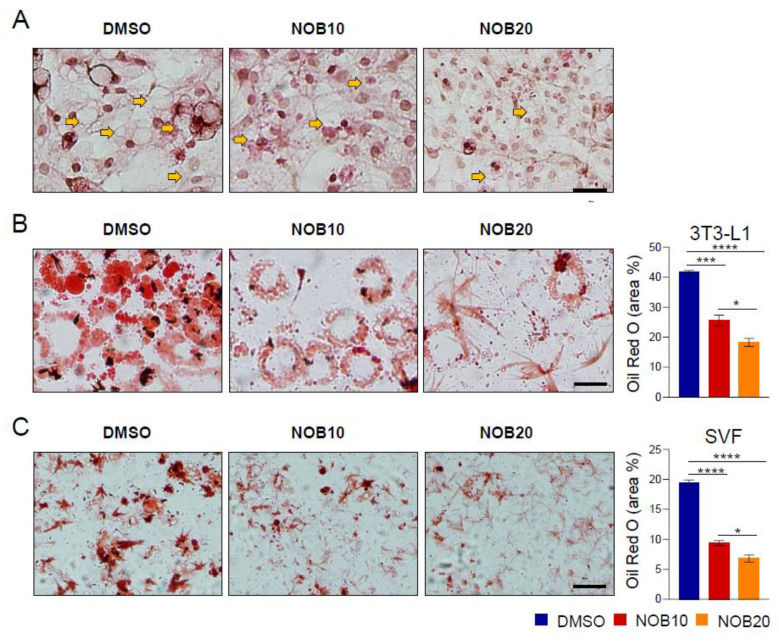
NOB inhibits lipid accumulation in differentiated 3T3-L1 and SVF cells. (**A**) Representative images of H&E staining of 3T3-L1 cells at 6 days after differentiation. Yellow arrows indicate representative lipid droplets. Scale bar = 200 µm (×10). (**B**) Representative images of Oil Red O staining of 3T3-L1 cells at 8 days after differentiation. Scale bar = 100 µm (×20). The right panel shows the quantification result. One-way ANOVA with Tukey’s multiple comparison tests (*, *p* < 0.05; ***, *p* < 0.001; ****, *p* < 0.0001). (**C**) Representative images of Oil Red O staining of SVF cells at 6 days after differentiation. Scale bar = 100 µm (×20). The right panel shows the quantification result. One-way ANOVA with Tukey’s multiple comparison test (*, *p* < 0.05; ****, *p* < 0.0001).

We next investigated the effects of NOB on lipid accumulation. In addition to 3T3-L1 cells, stromal vascular cell fraction (SVF) preadipocytes were also analyzed because the SVF in adipose tissues is enriched with adipocyte progenitor cells and was found to be controlled by the circadian clock [42,43]. Specifically, 3T3-L1 and SVF cells were treated with the complete induction medium containing insulin, IBMX, and DEX to induce their differentiation into mature adipocytes. NOB at 10 or 20 µM were also added starting with the complete induction media. On day 2 after induction, culture media were changed to DMEM supplemented with 10% FBS and 5 μg/mL of insulin, and the cells were cultured for another 6 days in the presence of NOB. H&E staining (Figure 2A) revealed that 3T3-L1 cells treated with DMSO exhibited a notable accumulation of lipid droplets, characterized by a larger size and broad distribution within the cells. In contrast, NOB treatment resulted in a reduction in the number and size of lipid droplets accumulated in adipocytes. Lipid accumulation was further measured by Oil Red O staining on day 8. NOB20 significantly suppressed lipid accumulation in 3T3-L1 (Figure 2B) and SVF (Figure 2C) adipocytes in a dose-dependent manner to 43.8% and 35.0%, respectively, compared to DMSO. These results show a strong effect of NOB to inhibit lipid storage in differentiated adipocytes.

### 3.3. NOB Decreases Transcription of Adipogenesis-Related Genes

**Figure 3:**
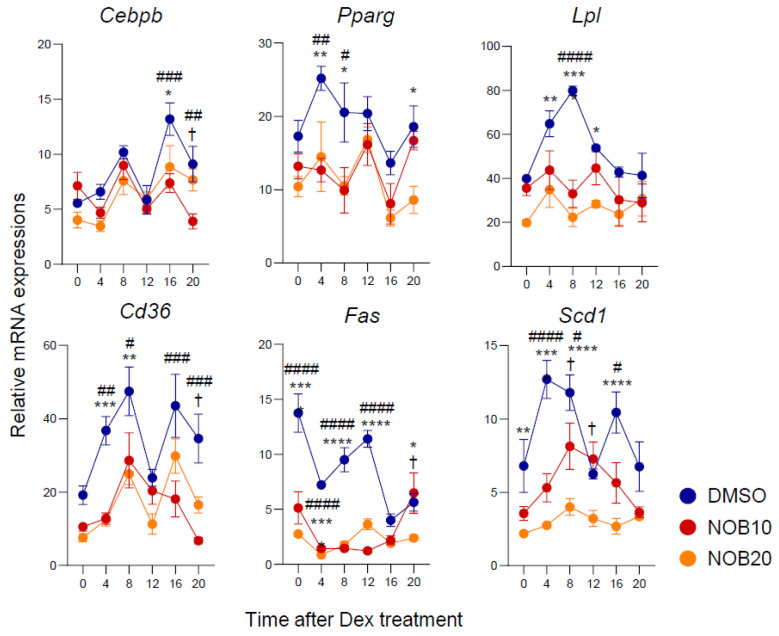
NOB reduces transcript levels of adipogenic differentiation-related genes. Real-time qPCR analysis of lipogenesis-related gene expressions in 3T3-L1 at 6 days after differentiation. Data are shown as mean ± SEM every 4 h for 24 h (*n* = 3/group/time point). Two-way ANOVA with Tukey’s multiple comparison tests showed significant differences # *p* < 0.05, ## *p* < 0.01, ### *p* < 0.001, #### *p* < 0.0001, DMSO vs. NOB10; * *p* < 0.05, ** *p* < 0.01, *** *p* < 0.001, **** *p* < 0.0001, DMSO vs. NOB20; † *p* < 0.05, NOB10 vs. NOB20.

Previous studies suggested a regulatory role of NOB in the adipogenesis and lipogenesis in 3T3-L1 cells [23,24,25]. To further investigate its effect on a circadian timescale, we conducted a qPCR analysis of key regulatory genes using 3T3-L1 cells collected over the circadian cycle. We observed that NOB decreased the transcript levels of genes associated with adipogenic differentiation throughout the circadian period (Figure 3). Specifically, expressions of adipogenesis-related genes such as *Cebpb* and *Pparg* and genes involved in lipid synthesis and accumulation such as *Lpl*, *Cd36*, *Fas*, and *Scd1* were diminished by NOB relative to DMSO, showing reduced expression levels and/or circadian amplitude. Together, these results indicate a strong effect of NOB to attenuate the circadian expression of genes involved in adipogenic differentiation and lipogenesis in 3T3-L1 cells.

### 3.4. NOB Regulates the IκBα/NF-κB Pathway and Inflammatory Cytokines

**Figure 4:**
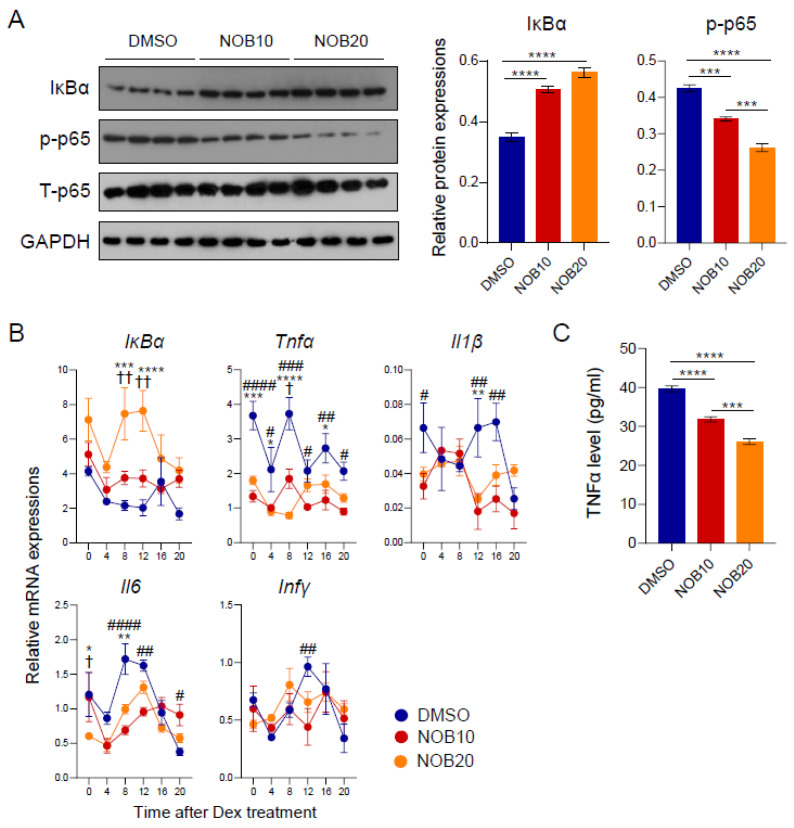
The IκBα/NF-κB pathway and inflammatory cytokines are regulated by NOB. (**A**) Protein expressions of IκBα and phosphorylation of p65 in 3T3-L1 at 8 days after differentiation. The right panel shows the quantification result. One-way ANOVA with Tukey’s multiple comparison test (***, *p* < 0.001; ****, *p* < 0.0001). (**B**) Real-time qPCR analysis of lipogenesis-related gene expressions in 3T3-L1 at 8 days after differentiation. Data are shown as mean ± SEM every 4 h for 24 h (*n* = 3/group/time point). Two-way ANOVA with Tukey’s multiple comparison tests showed significant differences # *p* < 0.05, ## *p* < 0.01, ### *p* < 0.001, ##### *p* < 0.0001, DMSO vs. NOB10; * *p* < 0.05, ** *p* < 0.01, *** *p* < 0.001, **** *p* < 0.0001, DMSO vs. NOB20; † *p* < 0.05, †† *p* < 0.01 NOB10 vs. NOB20. **(C**) TNFα level from media was measured by using ELISA. One-way ANOVA with Tukey’s multiple comparison test (***, *p* < 0.001; ****, *p* < 0.0001).

Inflammatory pathways, including NF-κB signaling, are aggravated in adipose tissues affected by obesity, resulting in the elevated expression of subsequent cytokines such as TNFα, IL6, IL1β, and others [10,11,12]. NF-κB regulation involves a crucial negative feedback loop achieved through the induction of IκBα expression directed by NF-κB itself [44,45]. Previously, we found that the NOB–ROR axis controls IκBα expression and subsequently attenuates NF-κB signaling in triple-negative breast cancer cells (TNBC) [22]. We therefore examined IκBα protein expression and the phosphorylation of p65, a key NF-κB subunit [46], in response to NOB in 3T3-L1 cells. Following NOB treatment, we observed that IκBα proteins were strongly induced, dose-dependently, in 3T3-L1 cells (Figure 4A). Furthermore, immunoblotting analysis showed that NOB treatment significantly attenuated levels of phosphor-p65 (Ser536) in a dose-dependent manner (Figure 4A), together indicating a conserved mode of action of NOB to inhibit NF-κB activation in multiple cell types.

To determine whether IκBα transcription is responsive to NOB in 3T3-L1 cells, we performed qPCR analysis and found that IκBα mRNA expression was activated by NOB, consistent with the above immunoblotting result (Figure 4B). Furthermore, known NF-κB target genes encoding pro-inflammatory cytokines, such as *Tnfα*, *Il1β*, and *Il6*, showed markedly reduced levels and an altered circadian phase following NOB treatment compared to DMSO. Finally, we measured TNFα levels secreted from adipocytes. TNFα is a pro-inflammatory cytokine and the first “adipokine” identified to be secreted by adipose tissue, and plays a key role in obesity-related metabolic disorders [47]. TNFα levels secreted from adipocytes were attenuated by NOB treatment in a dose-dependent manner (Figure 4C).

### 3.5. ROR-Dependent Circadian Gene Regulation Is Enhanced by NOB

Our prior research demonstrated that NOB activated RORs in the core circadian oscillator, influencing the expressions of both clock and clock-controlled genes (such as *Bmal1* and *Cry1*,) [27]. To examine the effects of the NOB–ROR axis on the circadian oscillator at the transcriptional levels, we generated Rora/Rorc double knockdown (Ror DKD) by CRISPR in 3T3-L1 cells as previously described [36] (Supplementary Figure S1). The cells were then synchronized by Dex and collected over the circadian cycle. qPCR analysis showed that Ror DKD cells displayed significantly altered clock gene expression patterns compared to the parental 3T3-L1 cells (Supplementary Figure S2). Interestingly, the expression of ROR target genes including *Bmal1*, *Cry1*, *Dec1*, *and Dec2* exhibited strongly decreased oscillation over the circadian cycle with generally lower expression levels in Ror DKD relative to 3T3-L1, and the activation effects of NOB were also attenuated in Ror DKD, suggesting an ROR-dependent NOB function. Together, these findings indicate a reduced circadian oscillation in Ror DKD cells, and the NOB–ROR axis significantly modulates the circadian expression of various ROR target genes.

### 3.6. RORs Are Required for NOB Efficacy to Mitigate Lipid Accumulation

**Figure 5:**
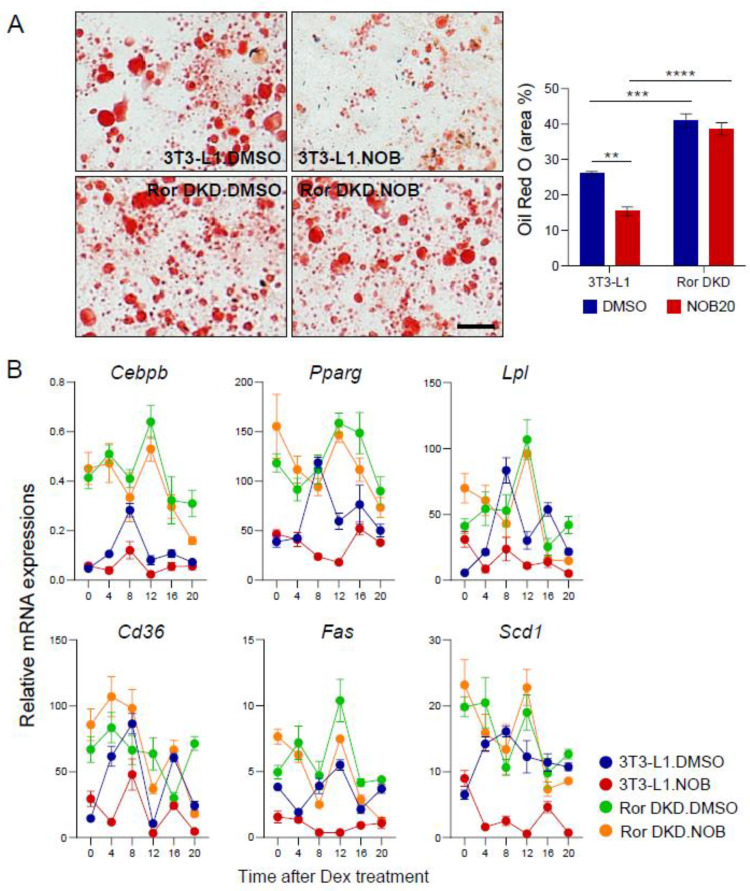
ROR is required for NOB-dependent mitigation of lipid accumulation. (**A**) Representative images of Oil Red O staining of Ror DKD 3T3-L1 cells treated with NOB 20 µM at 6 days after differentiation. Scale bar = 100 µm (×20). The right panel shows the quantification result. Two-way ANOVA with Tukey’s multiple comparison test (**, *p* < 0.01; ***, *p* < 0.001; ****, *p* < 0.0001). (**B**) Real-time qPCR analysis of adipogenic differentiation-related gene expressions at 6 days after differentiation. Data are shown as mean ± SEM every 4 h for 24 h (*n* = 3/group/time point).

RORα has been reported to negatively regulate adipocyte differentiation by inhibiting adipogenic gene expression and lipid accumulation [48,49,50]. In Ror DKD 3T3-L1 cells, we found that lipid accumulation was increased compared to 3T3-L1 cells (Figure 5A and Supplementary Figure S3). Through H&E staining, Ror DKO cells exhibited cavities within the cells due to the presence of lipid droplets. In Ror DKD cells, Oil Red O staining showed an exaggerated accumulation of lipid droplets within the adipocytes, with increases in both the number and size of lipid droplets compared to 3T3-L1 cells. Ror DKD cells also exhibited larger Oil Red O stain areas compared to 3T3-L1 cells, indicating attenuated NOB effects in the absence of RORs. Additionally, genes functionally involved in adipogenic differentiation, including *Cebpb*, *Pparg*, *Lpl*, *Cd36*, *Fas*, and *Scd1*, that were down-regulated by NOB were highly induced in Ror DKD cells, consistent with Oil Red O staining (Figure 5B). In particular, the circadian expressions of *Cebpb*, *Pparg*, and *Lpl* in Ror DKD cells displayed significant phase shifts compared to those in 3T3-L1 cells. While NOB decreased the expression of adipogenesis-related genes in 3T3-L1 cells, this effect was largely abrogated in Ror DKD cells.

### 3.7. The ROR–NOB Axis Targets the IκBα/NF-κB Pathway

**Figure 6:**
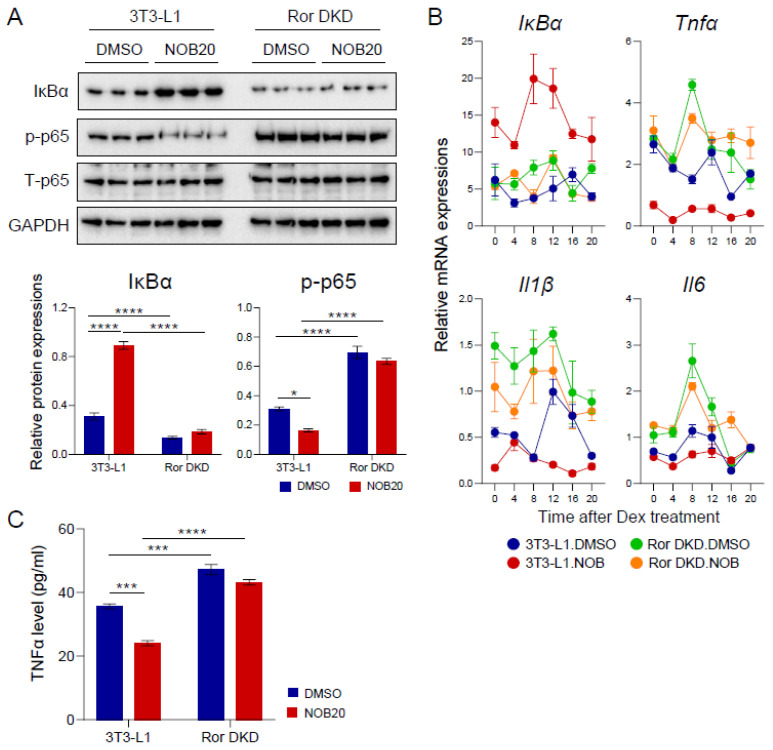
The IκBα/NF-κB pathway is a target of the ROR–NOB axis. (**A**) Protein expressions of IκBα and phosphorylation of p65 in differentiated 3T3-L1 cells treated with NOB 20 µM at 6 days after differentiation. The right panel represents the quantification result. Two-way ANOVA with Sidak’s (for IκBα) or Tukey’s (p-p65) multiple comparison test (*, *p* < 0.05; ****, *p* < 0.0001). (**B**) Real-time qPCR analysis of lipogenesis-related gene expressions at 6 days after differentiation. Data are shown as mean ± SEM every 4 h for 24 h (*n* = 3/group/time point). (**C**) TNFα level from media using ELISA. Data are shown as mean ± SEM. Two-way ANOVA with Tukey’s multiple comparison test (***, *p* < 0.001; ****, *p* < 0.0001).

Previous studies have identified IκBα, encoded by *NFKBIA*, as a direct transcriptional target of RORα in human primary smooth-muscle cells and TNBC cells [22,51]. To further investigate the role of RORs in IκBα/NF-κB signaling, we used parental 3T3-L1 and Ror DKD cells-treated NOB. As shown in Figure 6A, Ror DKD inhibited IκBα protein expression and induced the phosphorylation of p65 (also named RelA). NOB showed many diminished effects in Ror DKD cells in comparison with 3T3-L1 cells where NOB was able to significantly reduce p65 phosphorylation. These findings demonstrate that the effects of NOB on the IκBα/NF-κB pathway are dependent on RORs.

As expected, the induction of IκBα mRNA expression by NOB was diminished in Ror DKD (Figure 6B). Further qPCR analysis revealed that the inhibitory effects of NOB on proinflammatory cytokine gene expression were reversed in Ror DKD cells. In accordance, we observed that Ror DKD cells showed an elevated secretion of TNFα, and the effect of NOB was abolished in Ror DKD cells compared to 3T3-L1 cells (Figure 6C). These results indicate that ROR deletion counteracts NOB to regulate inflammation, providing critical mechanistic evidence linking NOB–ROR and IκBα/NF-κB signaling.

## 4. Discussions

The circadian clock has been shown to regulate cellular physiology in adipocytes, including trigyceride synthesis, storage, and triglyceride breakdown [35,52]. Here, we demonstrate that NOB, a natural flavonoid, enhanced the circadian oscillation of ROR-targeted genes and inhibited adipogenesis and lipogenesis in differentiated 3T3-L1 cells. This is achieved, at least in part, by increasing IκBα levels and suppressing p65 phosphorylation, leading to the downregulated expression of NF-κB target genes, including those encoding proinflammatory cytokines, and a reduction in TNFα secretion. Conversely, Ror DKD adipocytes exhibited NF-κB activation and elevated proinflammatory cytokine expressions, and the regulatory effects of NOB on the IκBα/NF-κB pathway were significantly attenuated in Ror DKD cells. These results highlight the important role of the NOB–ROR axis to regulate adipogenic differentiation and inflammation in 3T3-L1 cells.

Previous studies have demonstrated that RORs play significant roles in regulating tissue and systemic metabolism [33]. RORs interact with diverse endogenous and exogenous ligands, resulting in a wide array of physiological effects including metabolism and immunity [27,53,54,55,56,57]. Nobiletin (NOB), a natural compound, was initially identified as a powerful clock modulator by chemical screening, acting as a high-affinity ROR agonist [27]. The growing literature suggests a broad beneficial role of NOB against various chronic and age-related diseases, such as metabolic diseases and Alzheimer’s disease [27,28,30,31,58,59]. In this study, we demonstrate that the regulatory function of NOB in adipocytes is associated with circadian rhythms and requires RORs. We found that NOB increased the oscillation of core clock genes in differentiated adipocytes, with a notable impact on the changes in ROR target genes such as *Bmal1* and *Cry1*. Interestingly, Ror DKD cells displayed significantly reduced effects of NOB, consistent with our previous study where Rorac double knockdown in C2C12 muscle cells led to diminished oscillation of core clock gene expressions such as *Bmal1*, *Per2*, *Cry1*, *Nr1dr*, and *Dbp* compared with WT C2C12 cells [28]. Furthermore, *Dec1* and *Dec2* have been shown to be direct RORs targets and are involved in suppressing adipogenic differentiation [41,60]. NOB increased the overall mRNA levels of *Dec1* and *Dec2*, but this effect was diminished in Ror DKD cells [41,60]. These results highlight the role of RORs in mediating NOB effects, and indicate key regulator targets functioning to modulate adipocyte metabolism.

Adipocyte differentiation involves a series of programmed changes in gene expression. Transcription factors such as C/EBPs and PPARs are involved in adipogenesis [61] and regulate the expression of many adipogenic differentiation-related proteins, including LPL, SCD1, and FAS required for lipogenesis [62]. Previously, NOB was shown to suppress lipid accumulation, starting at a concentration of 10 µM, without cell toxicity across the range of 10–100 µM during adipocyte differentiation [23]. NOB has also been suggested to attenuate adipogenic differentiation in 3T3-L1 cells by decreasing levels of various proteins, as mentioned above [23,24,25]. In our study, the treatment of differentiated 3T3-L1 cells with NOB at 10 µM and 20 µM resulted in the downregulation of adipocyte differentiation-related genes, including *Cebpb*, *Pparg*, *Lpl*, *Scd1*, *Fas*, and *Cd36*, as reported in previous studies. Importantly, however, we demonstrate here that these genes showed circadian expression patterns which were regulated by NOB, specifically involving the alteration of both phase and level during the circadian cycle. This study, for the first time, establishes a link between circadian gene regulation by NOB–ROR and its functional role in adipogenic differentiation. Ongoing studies in the lab are investigating this function in vivo.

We further report that the effects of NOB in adipocytes were significantly reduced in cells where RORα and RORγ were knocked down (Ror DKD), indicating that the regulatory role of NOB on adipogenesis and lipogenesis is dependent on RORs. RORα is a potent regulator of adipocyte differentiation and glyceroneogenesis in adipocytes [48,49,50], hepatic glucose and lipid metabolism in the liver [50,63], and lipogenesis and cholesterol efflux in the skeletal muscle [64,65]. In particular, over-expressed Rorα4 in 3T3-L1 cells led to lower mRNA expressions of *Pparg*, *Cebpa*, *Ap2*, and *Srebp1c* [48]. On the other hand, mouse embryonic fibroblasts (MEFs) isolated from the *Staggerer* mice harboring a *Rora*-dominant negative mutation manifested higher expressions of *Pparg*, *Cebpa*, and *Srebp1c* than WT mice [48], and liver-specific *Rora* KO mice developed steatosis with elevated expressions of *Fas*, *Scd1*, *Acc*, and *Srebp1c* [63]. The RORγ function in adipose tissue is not well-studied, although it has been shown to be induced during the differentiation of 3T3-L1 cells [66]. Interestingly, the levels of RORγ detected in differentiated cells were approximately 10 times higher than those of RORα [66]. There were no significant differences in the overall body weight or serum triglycerides between WT and *Rorc^−/−^* mice, although a direct measurement of body mass composition was not conducted [67]. This suggests that the metabolic phenotype associated with the absence of RORγ may be milder compared to the effects observed in the absence of functional RORα. However, further investigation is needed to fully understand the specific role of RORs in the adipose tissue metabolism. Our study reveals the significant role of RORs in the lipid accumulation of adipocytes by suppressing the circadian expression of adipogenic differentiation-related genes, and highlights a functional requirement of RORs in the NOB-mediated effects in 3T3-L1 cells.

Obesity is a known risk factor for metabolic diseases and is often associated with chronic inflammation in adipose tissue. Anti-inflammatory effects of NOB have been reported in several models including macrophages [18,68], metabolic diseases [69,70], cancer [22,71], colitis [72,73], Alzheimer’s disease [30], and others. Furthermore, RORα1 plays a crucial regulatory role in the transcriptional activation of IκBα, the primary inhibitor of the NF-κB signaling pathway [51]. Consequently, this regulation leads to a reduction in p65 nuclear translocation and contributes to the attenuation of the inflammatory response induced by cytokines such as TNFα [51]. Additionally, our previous study revealed a direct transcriptional regulation of the IκBα gene promoter by the NOB–ROR axis, where NOB treatment enhances ROR promoter recruitment on the IκBα gene [22]. We also demonstrated that NOB–ROR plays a crucial role in suppressing TNFα-induced p65 phosphorylation and its subsequent nuclear localization [22,51]. These findings collectively offer valuable mechanistic insights into the impact of the NOB–ROR axis on cancer and inflammation [22]. However, the potential effects of NOB–ROR on inflammation in adipocytes and adipose tissue were largely unknown. In this study, NOB was found to increase the levels of IκBα protein and mRNA in differentiated 3T3-L1 cells, while significantly reducing the phosphorylation of p65. As a result, the mRNA levels of the NF-κB target genes encoding proinflammatory cytokines were decreased, and the secretion of TNFα, a pivotal proinflammatory cytokine and adipokine, from adipocytes was also downregulated by NOB treatment. On the other hand, Ror DKD adipocytes showed an activation of the NF-κB pathway, leading to elevated proinflammatory cytokine expression. Notably, the regulatory effect of NOB on the IκBα/NF-κB pathway observed was markedly attenuated in Ror DKD cells, suggesting a codependency of NOB and RORs in regulating inflammation in adipocytes.

In conclusion, this study elucidates a NOB–ROR axis that broadly modulates circadian gene expression and targets the IκBα/NF-κB signaling pathway, leading to a reduction in adipogenic differentiation. These results reveal an important role of the circadian machinery in the regulation of adipogenesis and inflammation in adipocytes, suggesting a chronotherapeutic approach toward the treatment of obesity. Future studies should investigate the therapeutic potential of NOB–ROR, toward the ultimate goal of a clock-based strategy to combat obesity and related metabolic disorders.

## Figures and Tables

**Table 1:** Primer sequences for RT-qPCR.

	Forward (5′-3′)	Reverse (5′-3′)
*Bmal1*	CCACCTCAGAGCCATTGATACA	GAGCAGGTTTAGTTCCACTTTGTCT
*Cry1*	CTGGCGTGGAAGTCATCGT	CTGTCCGCCATTGAGTTCTATG
*Per2*	ATGCTCGCCATCCACAAGA	GCGGAATCGAATGGGAGAAT
*Dbp*	CTGGCCCGAGTCTTTTTGC	CCAGGTCCACGTATTCCACG
*Nr1d1*	CATGGTGCTACTGTGTAAGGTGTGT	CACAGGCGTGCACTCCATAG
*Dec1*	CATGAGAACACTCGGGACC	CCACACGATGGAGATGAGTG
*Dec2*	AAACCTGCGCCAAAGAAGT	CTGGGTGTCCAGCTCTCAA
*C/ebpβ*	AAGCTGAGCGACGAGTACAAGA	GTCAGCTCCAGCACCTTGTG
*Pparγ*	GAAAGACAACGGACAAATCACC	GGGGGTGATATGTTTGAACTTG
*Lpl*	GGGAGTTTGGCTCCAGAGTTT	TGTGTCTTCAGGGGTCCTTAG
*Cd36*	AAGCTATTGCGACATGATT	GATCCGAACACAGCGTAGAT
*Fas*	GCAAATGAATGGGGGTACA	CAGTGTTCACAGCCAGGAGA
*Scd1*	CTGACCTGAAAGCCGCGAAG	GCGTTGAGCACCAGAGTGTA
*IkBα*	TCCTGAGCTCCGAGACTTTC	GCGTCAAGACTGCTACACTG
*Tnfα*	CACCACCATCAAGGACTCAA	TCCAGCCTCATTCTGAGACA
*Il1β*	TGTGGCAGCTACCTGTGTCT	TCATCTCGGAGCCTGTAGTG
*Il6*	ACAACCACGGCCTTCCCTACTT	CACGATTTCCCAGAGAACATGTG
*Infγ*	TCAAGTGGCATAGATGTGGAAGAA	TGGCTCTGCAGGATTTTCATG
*Gapdh*	CAAGGTCATCCATGACAACTTTG	GGCCATCCACAGTCTTCTGG

## Data Availability

The data presented in this study are available upon request to the corresponding authors.

## Supplementary Materials

The following supporting information can be downloaded at: https://www.mdpi.com/article/10.3390/nu15183919/s1, Supplementary Figure S1: Ror DKD cells were generated by CRISPR; Supplementary Figure S2: ROR-dependent circadian gene expression was enhanced by NOB. Supplementary Figure S3: Ror DKD cells showed accumulation of lipid droplets.
