# Supplementary material for: The Circadian Nobiletin-ROR Axis Suppresses Adipogenic Differentiation and IκBα/NF-κB Signaling in Adipocytes
Source: Nutrients. 2023 Sep 9;15(18):3919. doi: 10.3390/nu15183919 (PMC10537147; doi:10.3390/nu15183919)
Supplement: Supplementary file 1 [file nutrients-15-03919-s001.zip › nutrients-2592629-supplementary.pdf]

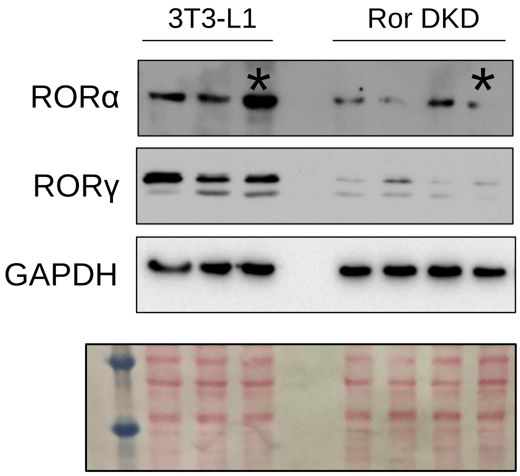

Supplementary Figure S1. Ror DKD cells were generated by CRISPR. Validation of RORα and RORγ protein expressions in Rorac KD cells. * indicated the cell clones used for further experiments. Lower panel; Ponceau staining for the membrane used in Western blotting.

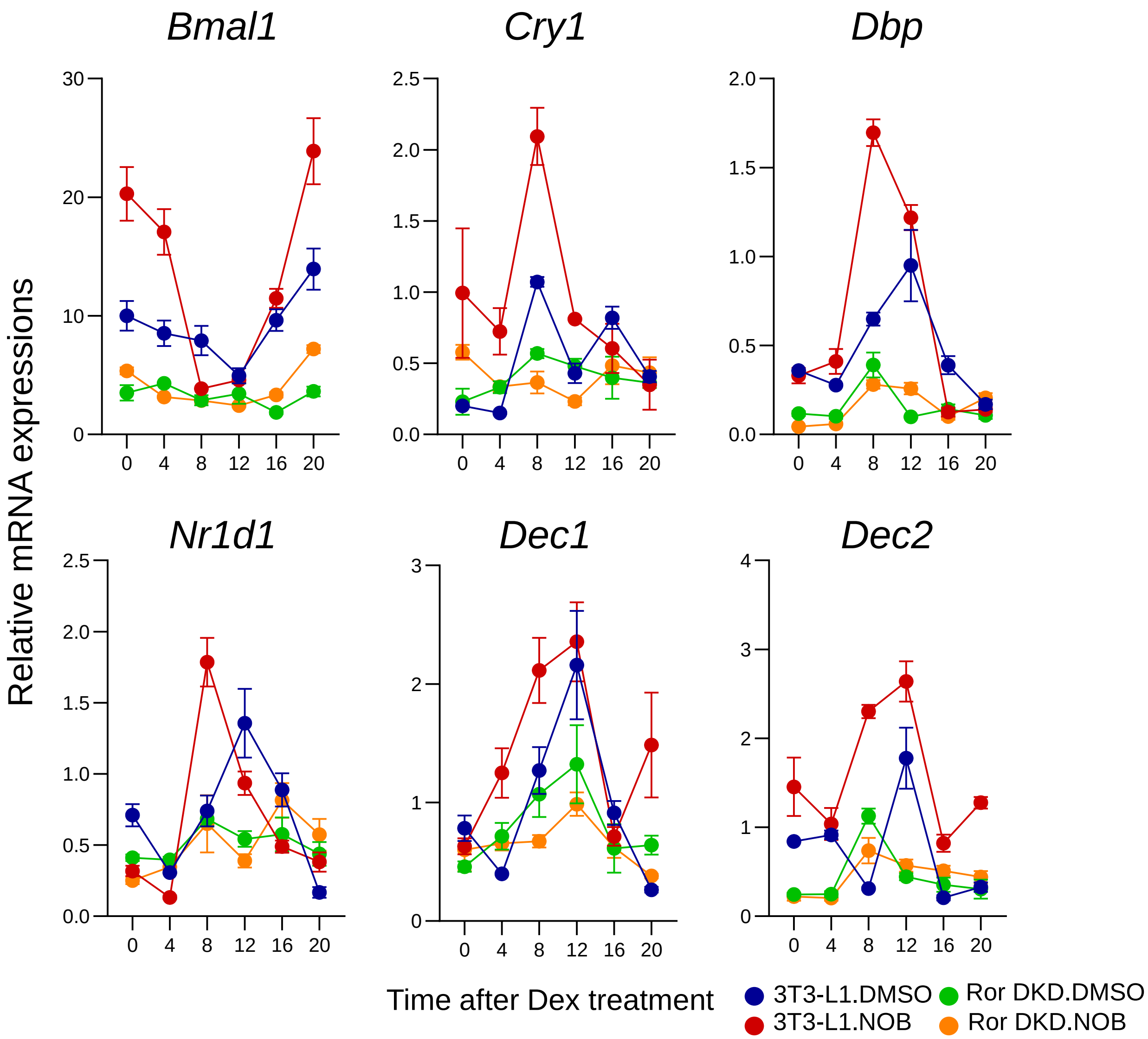

Supplementary Figure S2. ROR-dependent circadian gene expression was enhanced by NOB. A. Real-time qPCR analysis of core clock gene expressions of Ror DKD 3T3-L1 cells treated with NOB 20 μM at 6 days after differentiation. Data are shown as mean ± SEM every 4 h for 24 h (*n*=3/group/time point).

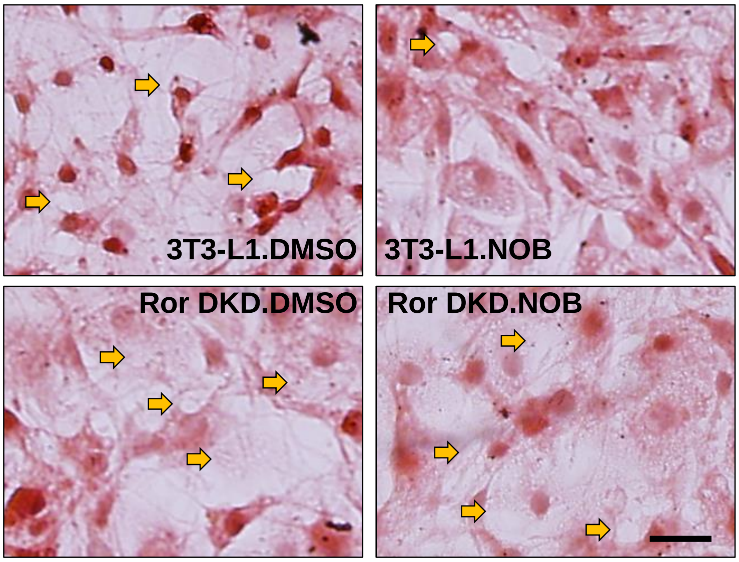

Supplementary Figure S3. Ror DKD showed accumulation of lipid droplets. Representative images of H&E staining of 3T3-L1 and Ror DKD cells treated with NOB 20 μM at 6 days after differentiation. Yellow arrows indicate representative lipid droplets. Scale bar = 200 μm (× 10).
